# Glutathione peroxidase 2 knockdown suppresses gastric cancer progression and metastasis via regulation of kynurenine metabolism

**DOI:** 10.1038/s41388-023-02708-4

**Published:** 2023-05-03

**Authors:** Handong Xu, Can Hu, Yi Wang, Yunfu Shi, Li Yuan, Jingli Xu, Yanqiang Zhang, Jiahui Chen, Qin Wei, Jiangjiang Qin, Zhiyuan Xu, Xiangdong Cheng

**Affiliations:** 1 Department of Gastric Surgery, Zhejiang Cancer Hospital, Hangzhou Institute of Medicine (HIM), Chinese Academy of Sciences, Hangzhou, 310022 China; 2 First Clinical Medical College, Zhejiang Chinese Medical University, Hangzhou, 310053 China; 3 Key Laboratory of Prevention, Diagnosis and Therapy of Upper Gastrointestinal Cancer of Zhejiang Province, Hangzhou, 310022 China; 4 Zhejiang Provincial Research Center for Upper Gastrointestinal Tract Cancer, Zhejiang Cancer Hospital, Hangzhou, 310022 China; 5 Tongde Hospital of Zhejiang Province, Hangzhou, China; 6 Key Laboratory of Cancer Prevention and Therapy Combining Traditional Chinese and Western Medicine of Zhejiang Province, Hangzhou, China

**Keywords:** Gastric cancer, Tumour biomarkers

## Abstract

Gastric cancer (GC) is among the most lethal malignancies due to its poor early diagnosis and high metastasis rate, and new therapeutic targets are urgently needed to develop effective anti-GC drugs. Glutathione peroxidase-2 (GPx2) plays various roles in tumor progression and patient survival. Herein, we found that GPx2 was overexpressed and negatively correlated with poor prognosis by using clinical GC samples for validation. GPx2 knockdown suppressed GC proliferation, invasion, migration and epithelial-mesenchymal transition (EMT) in vitro and in vivo. In addition, proteomic analysis revealed that GPx2 expression regulated kynureninase (KYNU)-mediated metabolism. As one of the key proteins involved in tryptophan catabolism, KYNU can degrade the tryptophan metabolite kynurenine (kyn), which is an endogenous ligand for AhR. Next, we revealed that the activation of the reactive oxygen species (ROS)-mediated KYNU-kyn-AhR signaling pathway caused by GPx2 knockdown was involved in GC progression and metastasis. In conclusion, our results showed that GPx2 acted as an oncogene in GC and that GPx2 knockdown suppressed GC progression and metastasis by suppressing the KYNU-kyn-AhR signaling pathway, which was caused by the accumulation of ROS.

## Introduction

Gastric cancer (GC) is the fifth most common malignancy and poses a significant global health burden. The International Agency for Research on Cancer (IARC) reported that approximately 1.09 million people were diagnosed with GC, and approximately 0.77 million deaths were attributed to GC in 2020 [1]. Due to a lack of practical early diagnostic strategies, most GC patients are often diagnosed at an advanced stage with metastases in lymph nodes, distant organs, or both, resulting in an overall 5-year survival rate < 40% [2]. Thus, it is important to clarify the molecular mechanisms of GC tumorigenesis or metastatic spread and identify GC-associated genes for early detection or targeted treatment to improve the prognosis of GC patients.

Glutathione peroxidase (GPx) is a reactive oxygen species (ROS)-scavenging defense system that can deactivate ROS to restore intracellular redox homeostasis [3]. ROS are important byproducts of cell metabolism and play dual roles in tumor cells, either initiating/stimulating tumorigenesis and supporting the transformation/proliferation of cancer cells or causing cell death [4]. GPx2 (also known as GSHPx-GI), a Se-dependent GPx, is found in the digestive tract epithelium and helps maintain mucosal homeostasis by reducing peroxide in the gut [3]. GPx2 plays considerable roles in the development, progression and maintenance of tumors, including those of lung cancer, pancreatic cancer, glioblastoma and breast cancer [5–8]. High GPx2 expression levels were associated with poorer prognosis in non-small cell lung cancer, glioblastoma, castration-resistant prostate cancer and nasopharyngeal carcinoma [5, 7, 9, 10], while the opposite trend was observed in breast cancer, esophageal squamous cell carcinoma and bladder cancer [8, 11, 12]. However, the relationship between GPx2 and GC is not clear, and further exploration is needed.

Tryptophan (Trp) is an essential aromatic amino acid and is considered necessary for producing many metabolites. Trp catabolites are also confirmed to be associated with tumorigenesis, progression, metastasis, and the immune response to many cancers [13]. Kynurenine (kyn) is an important metabolite of Trp that can activate the aryl hydrocarbon receptor (AhR) to participate in critical biological processes, such as cell differentiation, cell apoptosis and cell metastasis. Dai et al. confirmed that kyn can promote the invasion, migration and the progression of epithelial-mesenchymal transition (EMT) by activating AhR in renal cell carcinoma [14]. Kyn can also facilitate PD-L1-mediated immune evasion and maintenance of stemness by activating AhR in colon cancer [15]. In addition, kynureninase (KYNU) plays an essential role in kynurenine metabolism. Overexpression of KYNU can promote changes in metabolic kyn in melanoma [16]. Yang et al. [17] confirmed that KYNU-overexpressing CAR-T cells showed an improved ability to kill cancer cells by degrading kyn in the immunosuppressive tumor microenvironment. In this study, we assessed the expression of GPx2 and its correlation with clinicopathologic factors and prognosis. We first found that KYNU was increased and AhR was deceased in GPx2-knockdown GC cells and that GPx2 knockdown inhibited GC progression and metastasis via KYNU-mediated kyn metabolism by causing the accumulation of ROS.

## Results

### GPx2 was overexpressed in GC tissues and associated with tumor metastasis and poor prognosis

**Fig. 1:**
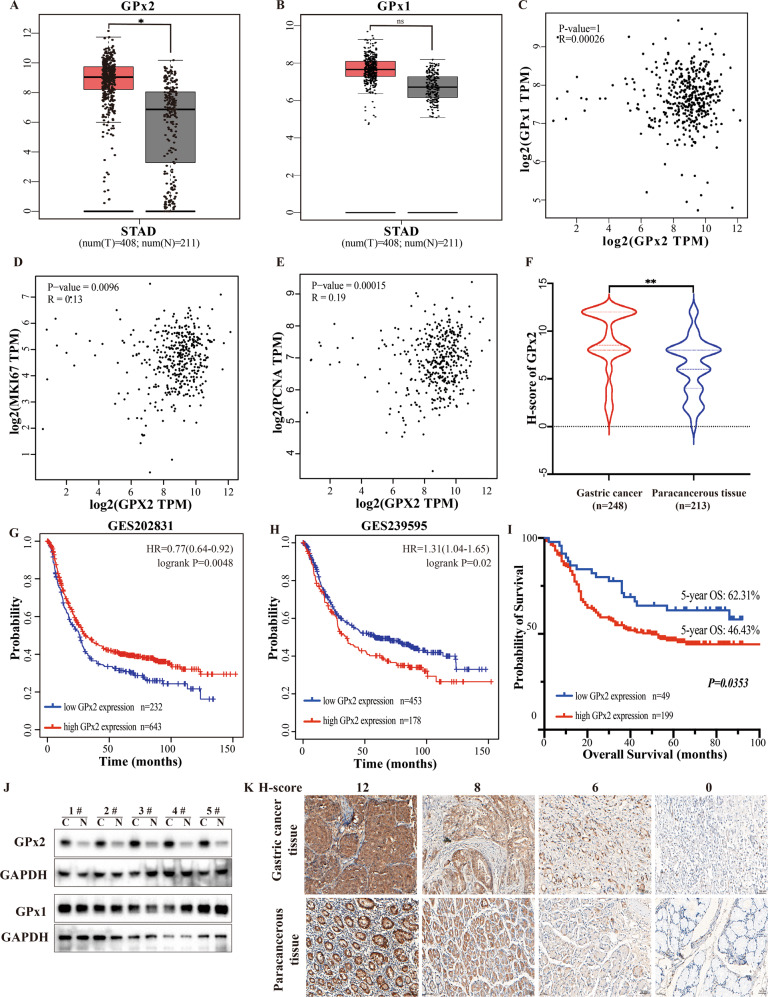
GPx2 is overexpressed in GC and correlated with poor prognosis. **A**, **B** GPx2 and GPx1 mRNA expression in GC in the GEPIA database. **C** The relationship between GPx2 mRNA expression and GPx1 mRNA expression in gastric cancer in the GEPIA database. **D**, **E** The relationship between GPx2 mRNA expression and KI67 or PCNA expression in the GEPIA database. **F** H-score of IHC showed GPx2 expression in gastric cancer tissues (*n* = 248) and paired paracancerous tissues (*n* = 213). **G**, **H** Survival analysis of the relationship between GPx2 expression and overall survival of GC patients in the GES 202831 (*n* = 875) and GES 239595 datasets (*n* = 631) in the Kaplan‒Meier Plotter database. **I** Survival analysis for the relationship between GPx2 expression and overall survival of GC patients in our cohort (*n* = 248). **J** Western blotting showing GPx2 and GPx1 protein expression in gastric cancer tissues and paired adjacent normal tissues (*n* = 5). **K** Representative IHC images of GPx2 expression in gastric cancer tissues and adjacent normal tissues (scale bar, 50 μm). *P < 0.05, **P < 0.01.

Using the GEPIA database [18] (GEPIA, http://gepia.cancer-pku.cn/), the mRNA expression levels of GPx2 and GPx1 in gastric cancer tissues and adjacent normal tissues were examined (Fig. 1A, B); GPx2 was overexpressed in GC tissues compared with adjacent normal tissues (*P* < 0.01), while there was no significant difference in GPx1 expression. In addition, there were no significant differences between GPx2 expression and GPx1 expression (*P* = 1, *R* = 0.00026) (Fig. 1C). Further analysis found that GPx2 mRNA expression was positively correlated with MKI67 mRNA expression (*P* = 0.0096, *R* = 0.13) and PCNA mRNA expression (*P* = 0.00015, *R* = 0.19) (Fig. 1D, E). Furthermore, we analyzed GPx2 expression in our GC TMAs by IHC. The GPx2 protein was found in the cytoplasm (Fig. 1K). However, the intensity of GPx2 staining was stronger in GC tissues than in paracancerous tissues. The median H-score of GPx2 expression was 8.9 in GC tissues, while the median H-score of GPx2 expression was 6.3 in paracancerous tissues (Fig. 1F). The median H-score of GPx2 expression was 6 (range: 0–12) in all samples, and the median score was used to determine the cutoff value of low or high GPx2 expression. An H-score ≤ 6.0 was defined as low GPx2 expression, and an H-scoreå 6.0 was defined as high GPx2 expression. A total of 199 (80.24%) patients showed high GPx2 expression, while 49 (19.76%) showed low GPx2 expression in GC tissues. In contrast, 109 (51.17%) paracancerous tissues showed high GPx2 expression, while 104 (48.83%) showed low GPx2 expression (Supplementary Table 1). In addition, we measured GPx2 and GPx1 expression in gastric cancer tissues and paired paracancerous tissues by Western blotting. The results showed that GPx2 was overexpressed in gastric cancer tissues compared with paired paracancerous tissues, while there was no significant difference in GPx1 expression between gastric cancer tissues and paired paracancerous tissues (Fig. 1J).

The effect of GPx2 expression on the prognosis of gastric cancer is still controversial. Survival analysis of GPx2 was performed with the GSE 202831 and GSE 239595 cohorts in the Kaplan‒Meier Plotter database (KM plotter, https://kmplot.com/analysis/index.php?p=service&cancer=gastric) [19] (Fig. 1H, I). Patients with low GPx2 expression had a poorer prognosis than those with high GPx2 expression in the GSE 202831 cohort (*P* = 0.0048), while patients with high GPx2 expression had a poorer prognosis than those with low GPx2 expression in the GSE 239595 cohort (*P* = 0.02). Next, we explored the relationship between GPx2 expression and clinicopathological characteristics in GC patients in our cohort. We found that there was a significant difference in 5-year OS between GC patients with high GPx2 expression and those with low GPx2 expression (46.43% vs. 62.31%, *P* = 0.035) (Fig. 1I). Moreover, the prognostic value of GPx2 expression in GC was investigated (Table 1), and the results showed that GPx2 overexpression was significantly correlated with N stage (*P* = 0.006) and KI67 expression (*P* = 0.029), while GPx2 expression was not significantly associated with age, sex, Lauren type, tumor size, T stage, M stage, TNM stage, CEA level or CA 199 level. These results suggest that GPx2 expression may be associated with lymph node metastasis and tumor cell proliferation.

**Table 1:** Correlations between GPx2 expression and clinicopathological characteristics of GC patients.

Variables	GPx2 expression		Total	χ^2^	*p*-value
	High	Low			
Age (year)					
≤65	120	33	153	0.826	0.364
>65	79	16	95		
Sex					
Female	58	16	74	0.231	0.631
Male	141	33	174		
Lauren type					
Intestinal GC	114	22	136	3.525	0.172
Diffuse GC	51	19	70		
Mixed GC	30	7	37		
Unknown	4	1	5		
Tumor size (cm)					
≤5 cm	92	21	113	1.593	0.451
>5 cm	104	28	132		
Unknown	3	0	3		
T stage					
T1 + T2	13	3	16	0.899	0.638
T3 + T4	184	46	230		
Unknown	2	0	2		
N stage					
N0 + N1	55	25	80	10.288	0.006*
N2 + N3	141	24	165		
Unknown	3	0	3		
M stage					
M0	176	45	221	1.166	0.558
M1	21	4	25		
Unknown	2	0	2		
TNM stage					
I + II	30	10	40	2.044	0.360
III + IV	166	39	205		
Unknown	3	0	3		
CEA (ng/ml)					
≤5	132	38	170	2.408	0.300
>5	49	8	57		
Unknown	18	3	21		
CA199 (U/ml)					
≤37	117	36	153	3.737	0.154
>37	64	10	74		
Unknown	18	3	21		
KI67					
Negative	79	25	104	7.113	0.029*
Positive	109	14	123		
Unknown	11	1	12		

^*^Statistically significant (*p* < 0.05).

### GPx2 expression promoted GC cell proliferation, invasion, migration and EMT in vitro

**Fig. 2:**
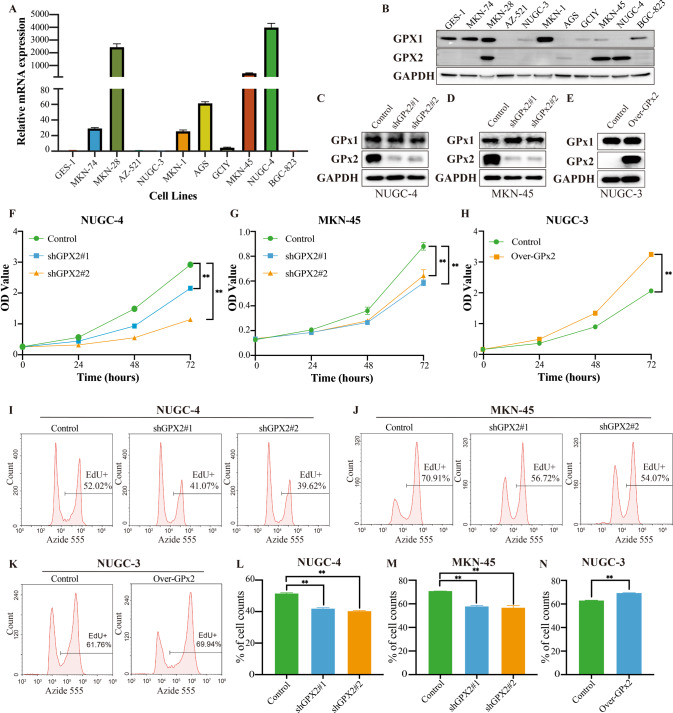
GPx2 expression promoted GC proliferation in vitro. **A**, **B** GPx2 mRNA and protein expression in GC cell lines. **C**, **D** Western blotting showing GPx2 and GPx1 expression after GPx2 knockdown in NUGC-4 and MNK-45 GC cell lines transfected with GPx2-shRNA and scrambled-shRNA. **E** Western blotting showing GPx2 and GPx1 expression in NUGC-3 GC cells overexpressing GPx2 and controls. **F**, **G** Results of CCK-8 assays following GPx2 knockdown. **H** Results of CCK-8 assays following GPx2 overexpression. **I**, **J** EdU analysis following GPx2 knockdown (The peak below 10^4^ of Azide 555 represents EdU-negative cells, the peak below 10^6^ of Azide 555 represents EdU-positive cells). **K** EdU analysis following GPx2 overexpression (The peak below 10^4^ of Azide 555 represents EdU-negative cells, the peak below 10^6^ of Azide 555 represents EdU-positive cells). **L**–**N** Quantitation of the EdU analysis data. *P < 0.05, **P < 0.01.

We analyzed the expression of GPx2 in various GC cell lines and a human gastric epithelial cell line (GES-1) by RT‒qPCR and Western blotting. The results revealed that MKN-28 GC cells, NUGC-4 GC cells and MKN-45 GC cells highly expressed GPx2 (Fig. 2A, B). The NUGC-4 and MKN-45 GC cell lines were derived from patients with gastric signet-ring cell carcinoma, while MKN-74, MKN-28, AZ-521, NUGC-3, MKN-1, AGS, GCIY, HGC-27, and BGC-823 were derived from patients with gastric adenocarcinoma. Gastric signet-ring cell carcinoma is a kind of low differentiated adenocarcinoma with strong invasion and metastasis abilities. In addition, the patients with NUGC-4 and MKN-45 GC cell lines also had liver metastasis. As suggested by the clinical data, we speculate that GPx2 expression may play an essential role in GC prognosis and progression. Subsequently, we established stable GPx-2 knockdown NUGC-4 and MKN-45 GC cells by transfection with GPx2-specific shRNA and established stable NUGC-3 GC cells that overexpressed GPx2 (Fig. 2C, D). Next, CCK-8 assays (Fig. 2F–H) and EdU incorporation assays (Fig. 2I–N) confirmed that downregulating GPx2 expression significantly inhibited proliferation, while GPx2 overexpression promoted the proliferation of GC cell lines.

**Fig. 3:**
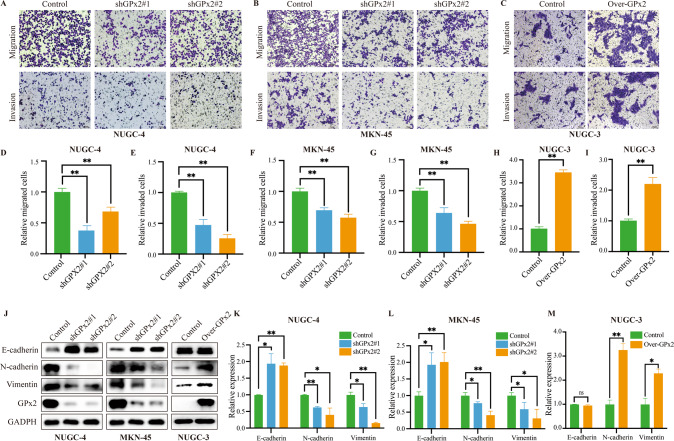
GPx2 expression promoted GC invasion, migration and EMT in vitro. **A**, **B** Results of transwell assays following GPx2 knockdown (scale bar, 50 μm). **C** Results of transwell assays following GPx2 overexpression (scale bar, 50 μm). **D**–**I** Quantitation of the data of transwell assays. **J** E-cadherin, N-cadherin, vimentin and GPx2 expression was detected by Western blotting following GPx2 knockdown or overexpression in GC cell lines. **K**–**M** Quantitation of the Western blotting data. *P < 0.05, **P < 0.01.

In addition, transwell assays showed that the migration and invasion abilities of GPx2-knockdown NUGC-4 and MKN-45 cells were significantly decreased, while those of GPx2-overexpressing NUGC-3 cells were increased (Fig. 3A–I). Epithelial-mesenchymal transition (EMT) is an essential biological process that plays an important role in tumor invasion and metastasis. Then, we evaluated the effect of GPx2 expression on EMT-associated proteins, such as E-cadherin (E-cad), N-cadherin (N-cad) and Vimentin. The results showed that the E-cad protein level was increased and the protein levels of N-cad and Vimentin were decreased in GPx2-silenced GC cells, while the opposite trend occurred when GPx2 was overexpressed (Fig. 3J–M). Therefore, GPx2 expression may be associated with the proliferation, migration, invasion and EMT of GC cells.

### Proteomic analysis revealed that GPx2 expression can regulate KYNU-mediated metabolism

**Fig. 4:**
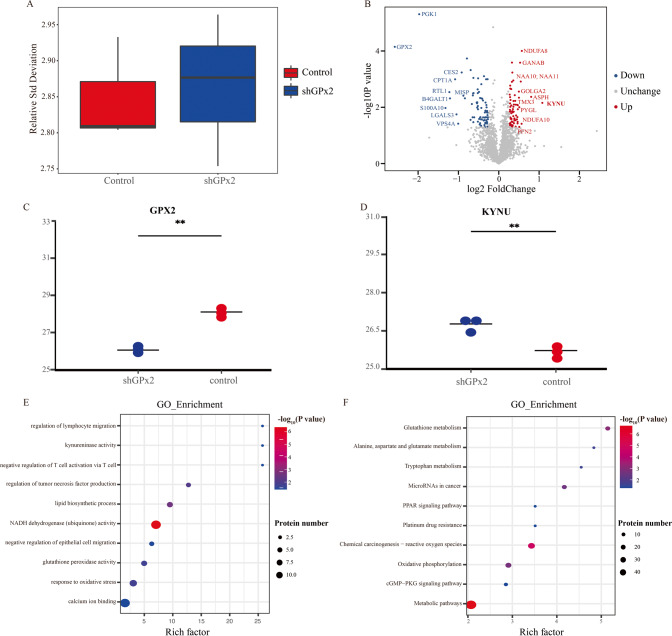
Proteomic analysis of NUGC-4 GC cells with GPx2 knockdown. **A** RSD analysis of mass spectrum results. **B** Volcano plot showing the differentially expressed proteins in NUGC-4 cells following GPx2 knockdown. **C**, **D** GPx2 and KYNU expression in proteomic analysis following GPx2 knockdown. **E**, **F** GO analysis and KEGG analysis of the significantly differentially expressed proteins in NUGC-4 cells following GPx2 knockdown. *P < 0.05, **P < 0.01.

To elucidate the molecular mechanism by which GPx2 promotes the progression of GC, quantitative proteome analysis was performed with NUGC-4 GC cells and GPx2-knockdown NUGC-4 GC cells. Relative standard deviation (RSD) analysis was performed for quality control of the mass spectrum results (Fig. 4A). After preliminary screening, a total of 147 genes (fold change ≥ 1.2, *P* < 0.05) were identified as differentially expressed proteins. Compared with the control group, 69 genes were upregulated and 78 genes were downregulated in the shGPx2 group (Fig. 4B, Supplementary Table 2). GPx2 and KYNU were the two genes most differentially expressed (Fig. 4C, D). To understand the effect of differentially expressed proteins in GC cells, KEGG pathway and GO enrichment analyses were performed. GO analysis showed that the differentially expressed proteins after GPx2 knockdown were mainly involved in the regulation of lymphocyte migration, kynureninase activity, regulation of T-cell activation, regulation of tumor necrosis factor production, lipid biosynthetic process, regulation of epithelial cell migration, glutathione peroxidase activity and response to oxidative stress (Fig. 4E), while KEGG analysis showed that GPx2 expression could regulate glutathione metabolism, tryptophan metabolism, the PPAR signaling pathway, oxidative phosphorylation and the cGMP-PKG signaling pathway (Fig. 4F).

### GPx2 knockdown inhibited GC cell invasion and metastasis via the KYNU-mediated kyn-AhR signaling pathway

**Fig. 5:**
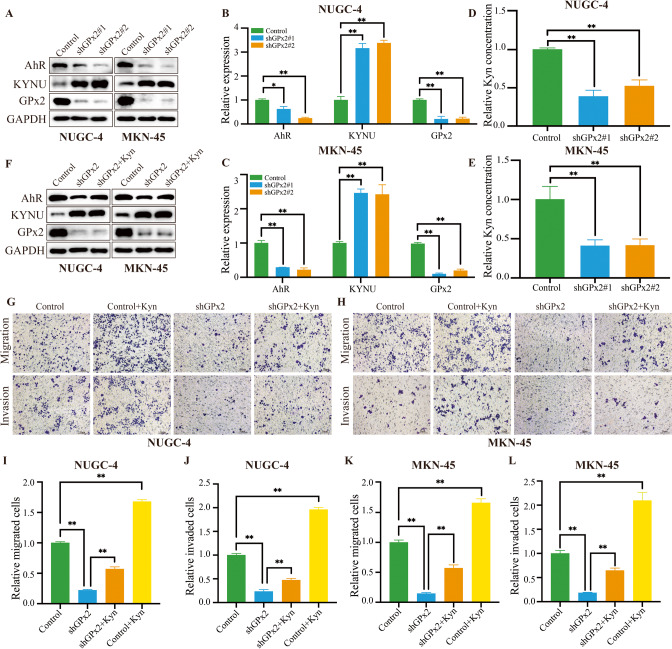
GPx2 knockdown suppressed GC invasion and migration by promoting KYNU-mediated kynurenine metabolism. **A** GPx2, KYNU and AhR expression was detected by Western blotting following GPx2 knockdown. **B**, **C** Quantitation of the Western blotting data. **D**, **E** Results of ELISA following GPx2 knockdown. **F** GPx2 and AhR expression was detected by Western blotting after treatment with kyn (150 μM) for 24 h in GPx2 knockdown GC cell lines. **G**, **H** Transwell assays were performed after treatment with kyn (150 μM) or PBS for 24 h in GPx2 knockdown GC cell lines (scale bar, 50 μm). **I**–**L** Quantitation of the data from transwell assays. *P < 0.05, **P < 0.01.

Based on the proteomic results, we further verified the expression of KYNU after GPx2 knockdown using Western blotting. Consistent with the results of quantitative proteomic analysis, KYNU expression was upregulated after GPx2 knockdown in NUGC-4 GC cells and MKN-45 GC cells (Fig. 5A–C). As one of the key proteins involved in tryptophan catabolism, KYNU can degrade the tryptophan metabolite kyn, which is an endogenous ligand for AhR. Therefore, we analyzed AhR expression and kyn concentration after silencing GPx2 expression in NUGC-4 GC cells and MKN-45 GC cells. The results showed that AhR expression was downregulated (Fig. 5A–C), and the kyn concentration was decreased after silencing GPx2 (Fig. 5D, E). Next, we further examined whether GPx2 expression promotes GC metastasis through kyn. The results showed that kyn could rescue AhR expression (Fig. 5F) and the function of inhibiting GC invasion and metastasis caused by GPx2 knockdown (Fig. 5G–L). These results indicated that the inhibition of GC cell invasion and migration by GPx2 knockdown may be related to the KYNU-mediated kyn-AhR signaling pathway.

### GPx2-mediated ROS levels can regulate the KYNU-mediated kyn-AhR signaling pathway in GC cells

**Fig. 6:**
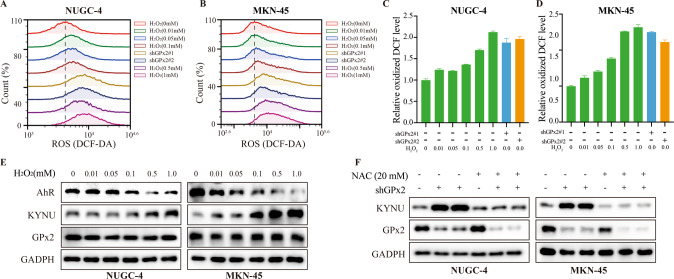
GPx2 knockdown promoted KYNU-mediated kyn metabolism by increasing ROS levels. **A**, **B** Flow cytometry assays following GPx2 knockdown or treatment with H_2_O_2_ (0.01, 0.05, 0.1, 0.5, and 1.0 mM) for 30 min. **C**, **D** Quantitation of the flow cytometry assay data. **E** GPx2, KYNU, and AhR expression was detected by Western blotting after treatment with H_2_O_2_ (0.01, 0.05, 0.1, 0.5, and 1.0 mM) for 30 min in GC cell lines. **F** GPx2 and KYNU expression was detected by Western blotting after treatment with NAC (20 mM) for 1 h in GC cell lines or GPx2 knockdown GC cell lines. *P < 0.05, **P < 0.01.

We further explored the mechanism by which GPx2 regulates KYNU expression. GPx2 is an important enzyme that can clear and deactivate ROS to restore intracellular redox homeostasis. Thus, we analyzed the ROS level after GPx2 knockdown or H_2_O_2_ treatment in NUGC-4 and MKN-45 GC cells. The results showed that the ROS level was increased after GPx2 knockdown or H_2_O_2_ treatment (Fig. 6A–D). We further hypothesized that ROS levels are correlated with KYNU expression. To directly assess whether ROS could regulate KYNU expression, we treated NUGC-4 and MKN-45 cells with H_2_O_2_. Interestingly, KYNU expression was increased, while AhR expression was decreased with H_2_O_2_ treatment in a concentration-dependent manner (Fig. 6E). Moreover, we also found that reducing the ROS level inhibited KYNU expression after N-acetyl-L-cysteamine (NAC) treatment (Fig. 6F). These results indicated that GPx2-mediated ROS levels could regulate the KYNU-mediated kyn-AhR signaling pathway in GC cells.

### GPx2 knockdown suppressed tumor proliferation and metastasis signaling in vivo

**Fig. 7:**
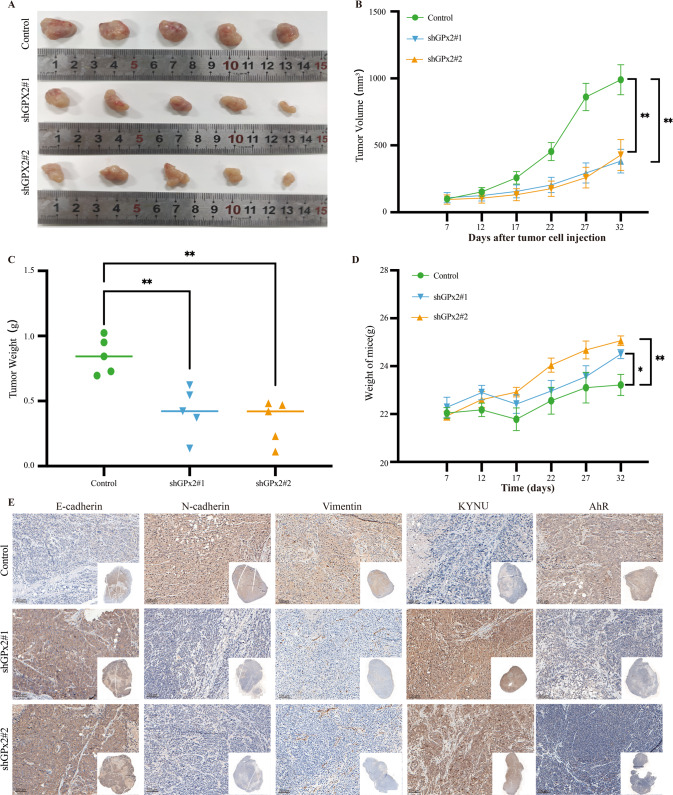
GPx2 knockdown suppressed GC proliferation in gastric xenograft tumor models. **A** GC cells stably transfected with GPx2 shRNA or empty vector were subcutaneously inoculated into nude mice. The mice were randomly divided into the control, shGPx2#1, and shGPx2#2 groups as described in the Methods. **B** The tumor sizes were determined using Vernier calipers. Tumor growth curves were generated based on the tumor volumes measured in the mice. **C** The tumor weights were determined at the end of the experiments. **D** The body weights of nude mice were determined every five days. E E-cadherin, N-cadherin, Vimentin, KYNU and AhR were detected by IHC (scale bar, 100 μm). *P < 0.05, **P < 0.01.

We established gastric xenograft tumors and peritoneal metastasis models to further determine whether GPx2 expression could promote GC proliferation and metastasis. NUGC-4 GC cells stably transfected with GPx2-shRNA or empty vector were subcutaneously inoculated into nude mice. We divided the mice into control, shGPx2#1, and shGPx2#2 groups. As shown in Fig. 7A–D, GPx2 knockdown suppressed tumor proliferation in gastric xenograft tumor models. Next, we examined the expression of KYNU, AhR and EMT-associated proteins in gastric xenograft tumors via IHC assay. The results showed that the expression of KYNU and E-cad in the shGPx2#1 and shGPx2#2 groups was higher than that in the control group, while N-cad, Vimentin and AhR expression was decreased in the shGPx2#1 and shGPx2#2 groups (Fig. 7E).

**Fig. 8:**
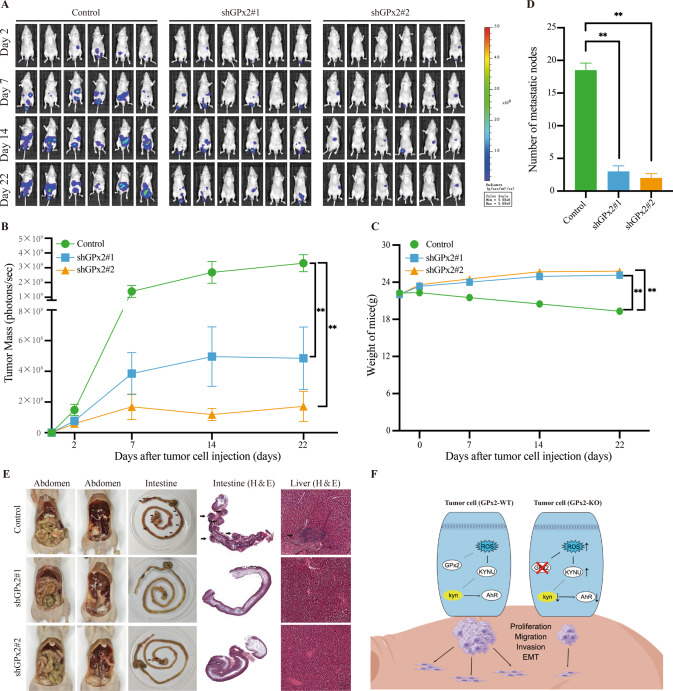
GPx2 knockdown suppressed GC metastasis in peritoneal metastasis models. **A** GC cells stably transfected with GPx2 shRNA or empty vector were intraperitoneally injected into nude mice. The mice were randomly divided into the control, shGPx2#1, and shGPx2#2 groups as described in the Methods. The luciferase signals in the mice were detected, and images were obtained using an IVIS imaging system. **B** The average tumor mass (determined by the detected photons/sec) of the mice in the control, shGPx2#1, and shGPx2#2 groups. **C** The body weights of nude mice were determined in the control, shGPx2#1, and shGPx2#2 groups. **D** Metastatic nodules of peritoneal metastasis in the control, shGPx2#1, and shGPx2#2 groups. **E** Peritoneal metastasis in the abdominal cavity, representative H&E image of intestine metastasis and liver metastasis in the control, shGPx2#1, and shGPx2#2 groups (black arrows represent metastatic nodules). **F** A working model for the role of GPx2 and the KYNU-kyn-AhR signaling pathway in regulating GC progression.

Furthermore, we assessed the metastatic effect of GPx2 expression in peritoneal metastasis models. GPx2 knockdown MKN-45 GC cells or control MKN-45 GC cells were injected into the abdominal cavity of mice, and the mice were administered fluorescein substrate (150 mg/kg) intraperitoneally for in vivo imaging once a week on a Xenogen IVIS 200 imaging system (Caliper Life Sciences, USA). The results were analyzed using the LT Living Image 4.3 Software, which confirmed that GPx2 knockdown significantly suppressed GC development and metastasis (Fig. 8A–C). Moreover, we counted the number of metastatic nodules in the peritoneal cavity, and the number of nodules was significantly decreased in the shGPx2#1 and shGPx2#2 groups compared with the control group (Fig. 8D). Furthermore, the H& E assay (Fig. 8E) also showed a similar result. Taken together, these results indicated that GPx2 knockdown suppressed GC progression and metastasis via the KYNU-mediated kyn-AhR signaling pathway in vivo.

## Discussion

Radical surgical resection with chemotherapy and/or radiotherapy is the primary treatment for GC [20]. However, because of the low number of early diagnoses, most GC patients are at an advanced stage with poor prognosis when first diagnosed. In recent years, targeted therapy and immunotherapy have developed rapidly in tumor therapy, but their efficacy in the treatment of GC is still not ideal. Therefore, new therapeutic targets are urgently needed to develop effective anti-GC drugs [21]. In the present study, we determined that GPx2 is highly expressed in GC tissues and is closely associated with tumor metastasis and survival outcomes in GC patients. Moreover, we also found that increased ROS levels caused by GPx2 knockdown can inhibit GC progression via the KYNU-mediated kyn-AhR signaling pathway. These findings not only enhance our understanding of the mechanisms underlying GC development but also reveal a key protein that may serve as a predictive biomarker and effective therapeutic target.

GPx is one of the most efficient antioxidants, and its main biological functions are to protect organisms from oxidative damage by reducing lipid hydroperoxides and free hydrogen peroxide [22]. GPx family proteins can protect against tumor initiation and carcinogenesis by preventing the accumulation of deleterious levels of oxidants that elicit macromolecular damage [23]. Conversely, many researchers have also suggested that many cancer cells require antioxidant enzyme upregulation for tumor progression and metastasis [24, 25]. GPx2 is found in epithelial tissues and digestive tract epithelium, and it may be a prognostic factor in cancers. However, its role in tumor development remains controversial [3]. Zuen et al. suggested that silencing GPx2 expression could increase the level of ROS, which in turn causes vascular malfunction and malignant progression [8]. In contrast, GPx2 was confirmed as an oncogene that could promote malignant progression and cisplatin resistance in non-small cell lung carcinoma (NSCLC) [5]. In addition, the increase in GPx2-mediated ROS levels could suppress tumor development via the Hippo pathway in lung squamous cell carcinoma [26]. Our study confirmed that GPx2 was overexpressed in GC tissues and was closely associated with tumor metastasis and survival outcomes in GC patients. Of note, among all the GC cell lines examined, GPx2 expression levels were particularly high in NUGC-4 and MKN-45 cells compared with GES-1 normal gastric epithelial cells, MKN-74, AZ-521, MKN-1, etc. Further analysis showed that the NUGC-4 and MKN-45 GC cell lines were derived from patients with gastric signet-ring cell carcinoma, which is a kind of low differentiated adenocarcinoma with strong invasion and metastasis abilities. This indicated that high GPx2 expression may be associated with invasion and metastasis. In addition, GPx2 knockdown significantly increased ROS levels in GC cells and suppressed GC progression and metastasis.

Next, we explored the mechanism by which GPx2 regulates GC progression and metastasis. Based on proteomic analysis, we found that GPx2 expression could regulate oxidative stress-related pathways and metabolic pathways. KYNU, as the most significantly affected protein after silencing GPx2 expression, was involved in tryptophan metabolism. Increased KYNU expression can reduce the amount of the tryptophan metabolite kyn, which plays a crucial role in regulating tumor development [27]. Kyn not only inhibits the anticancer immune response by inhibiting the proliferation of functional T cells [28] but also constitutively activates AhR, which has been confirmed as an effective contributor to tumor progression and EMT [29–31]. In our study, we confirmed that KYNU expression was increased after GPx2 knockdown in GC cells, which further decreased the kyn concentration to suppress AhR expression in vitro [32].

We further explored the mechanism by which GPx2 regulates KYNU expression. ROS, as one of the most important metabolites after GPx2 knockdown, modulates the tumor microenvironment, affecting various stromal cells that provide metabolic support, blood supply, tumor immune response and tumor metastasis [33]. In addition, ROS can also regulate some important signal transduction networks associated with tumor progression, such as the NF-kB signaling pathway and the PI3K/mTOR signaling pathway [34]. Therefore, we hypothesized that the increase in KYNU expression caused by GPx2 knockdown may be associated with the accumulation of ROS. As expected, KYNU expression was increased after GPx2 knockdown or exogenous H_2_O_2_ treatment, while clearing the ROS level inhibited KYNU expression after NAC treatment. Therefore, our results confirmed that GPx2 expression could promote GC progression and metastasis via the ROS-mediated KYNU-kyn-AhR signaling pathway.

## Conclusion

In this study, we identified a novel GC target, GPx2, that plays an important role in promoting GC progression and metastasis. We revealed that the accumulation of ROS caused by GPx2 knockdown can suppress GC progression and metastasis via the KYNU-kyn-AhR regulatory pathway. Our study not only provided evidence supporting the role of GPx2 in the progression of GC but also identified a novel potential prognostic marker and therapeutic target for GC.

## Materials and methods

### Cell lines and reagents

Human GC cell lines (NUGC-4, MKN-74, AZ-521, MKN-1, NUGC-3, AGS, HGC-27, MKN-45) were obtained from Shanghai Bioleaf Biotech Co., Ltd. (Shanghai, China). The human gastric epithelial cell line GES-1 was obtained from the Cell Bank of the Chinese Academy of Science (Shanghai, China). All cell lines were recently authenticated by short tandem repeat authentication and tested for mycoplasma contamination. NUGC-4, MKN-74, AZ-521, MKN-1, NUGC-3, HGC-27, MKN-45, and GES-1 cells were cultured in RPMI-1640 (BasalMedia, Shanghai, China), and AGS cells were cultured in Ham’s F12 (Cienry, Hu Zhou, China) containing 10% fetal bovine serum (FBS, Gibco, Grand Island, USA) and 1% penicillin/streptomycin (Kino Co., Ltd., Hangzhou, China) at 37 °C under 5% CO_2_ in a cell culture incubator [35]. NAC, a ROS scavenger, was purchased from Sigma–Aldrich (St. Louis, MO, USA). L-kyn was also purchased from Sigma‒Aldrich.

### Patients and clinicopathological characteristics

In this study, 269 patients who underwent gastrectomy were enrolled at Zhejiang Cancer Hospital from 2007 to 2017. Meanwhile, we collected demographic information and clinicopathological characteristics to analyze the relationship between those characteristics and GPx2 expression, including age, sex, Lauren type (a histo-clinical classification, which divides GC into intestinal GC, diffuse GC and mixed GC), tumor size, T stage (which reflects the depth of tumor infiltration), N stage (which reflects tumor lymph node metastasis), TNM stage (which refers to the eighth edition of the AJCC staging standard), CEA level, CA199 level, and KI67 expression.

### Tissue microarray (TMA) construction and immunohistochemistry (IHC) analysis

Primary GC tissues and adjacent noncancerous tissues from 269 patients who underwent gastrectomy were collected at Zhejiang Cancer Hospital from 2007 to 2017. Informed consent was obtained from all subjects. TMAs were constructed, including 248 GC tissues and 213 noncancerous tissues. Then, IHC staining and analysis were performed. IHC staining with antibodies against GPx2 (#ab140130, Abcam), KYNU (#11796-1-AP, Proteintech), AhR (#67785-1-Ig, Proteintech), E-cadherin (#3195 S, Cell Signaling Technology), N-cadherin (#13116 S, Cell Signaling Technology), and Vimentin (#5741 S, Cell Signaling Technology) was performed to measure protein expression levels using standard procedures. Protein expression was assessed using the H-score system. The formula for the H‐score was as follows: H‐score = ∑ (IS × AP), where IS represents the staining intensity (0, no staining; 1, weak staining; 2, intermediate staining; 3, strong staining) and AP represents the percentage of positively stained tumor cells (0, <5% of the total cells; 1, 5–25%; 2, 26–50%; 3, 51–75%; 4, 76–100%), producing an H-score ranging between 0 and 12. To assess the average degree of staining within a tumor sample, multiple regions were analyzed, and at least 100 tumor cells were assessed. Two experienced pathologists who were blinded to the clinical outcomes performed the scoring independently. The protocol was approved by the Committee on the Ethics of Zhejiang Cancer Hospital (IRB-2020-109).

### Generation of concentrated lentiviral vectors and infection

Lentivirus containing shRNA targeting GPx2 or GPx2 overexpression constructs or a negative control shRNA was synthesized by GeneChem Biotechnology Company (Shanghai, China). The sequences of 21 nucleotide shRNAs targeting GPx2 were CCGATCCCAAGCTCATCATTT and GCGCCTCCTTAAAGTTGCCAT. GC cells were transfected with lentivirus according to the manufacturer’s instructions. After 72 h, stable cell lines were screened using 1 µg/ml puromycin. Transfection efficiency was determined by Western blotting.

### RNA isolation and quantitative RT‒PCR

Total RNA was extracted using the RNA-Quick Purification Kit (Yishan Biotech, Shanghai, China) and reverse transcribed by using a ReverTra Ace qPCR RT kit (Toyobo). Subsequently, qPCR was performed using SYBR Green reagent (CWBIO) on a CFX96 Touch Real-Time PCR Detection System (Bio-Rad). The relative gene expression was calculated using the 2^−^ΔΔCq method. The primers used in the present study were as follows: GAPDH forward, AACGGATTTGGTCGTATTG and reverse, GGAAGATGGTGATGGGATT; GPx2 forward, CCCTCATGACCGATCCCAAG and reverse, TCCGGCCCTATGAGGAACTT.

### Quantitative proteome analysis

Stable control and shGPx2 cells were collected and sent to GeneChem Biotechnology Company (Shanghai, China) for quantitative proteome library construction and sequencing. Then, protein extraction, protein quantification, protein enzymatic hydrolysis and mass spectrometry detection were performed. The MS data were analyzed using MaxQuant software (version 1.6.17.0). MS data were searched against the database. The cutoff of the global false discovery rate (FDR) for peptide and protein identification was set to 0.01. Protein abundance was calculated on the basis of the normalized spectral protein intensity (LFQ intensity). Proteins with a fold change > 1.5 and *P*-value (Student’s t-test) <0.05 were considered to be differentially expressed [36]. The differentially expressed proteins were selected for Gene Ontology (GO) enrichment and Kyoto Encyclopedia of Genes and Genomes (KEGG) enrichment analysis.

### Cell viability assay

CCK-8 (GLPBIO, United States) assays were conducted to measure cellular viability. The transfected NUGC-4, MKN-45 and NUGC-3 cell lines were seeded into 96-well culture plates and incubated with CCK-8 reagent for 3 h. Thereafter, a microplate reader (Thermo Varioskan LUX, MA, United States) was used to measure the absorbance (OD) at 450 nm.

### EdU incorporation assay

EdU incorporation was measured with the BeyoClick™ EdU Cell Proliferation Kit with Alexa Fluor 555. Transfected GC cells were seeded into 12-well plates. After 24 h of incubation, the cells were incubated with 10 µM EdU (Beyotime, Shanghai, China) for 2 h. Then, the cells were harvested and fixed with 4% paraformaldehyde. After washing and permeabilizing, the cells were incubated with Click-iT® EdU reaction cocktail for 30 min. After washing, the EdU-positive cells were measured by flow cytometry (Agilent).

### Transwell migration and invasion (Matrigel) experiments

For migration assays, 1 × 10^5^ cells in 200 µL of serum-free media were seeded in the upper chamber of an insert (8 µm pore size, Corning, USA). For invasion assays, 1 × 10^5^ cells in 200 µL of serum-free media were seeded in the upper chamber of an insert coated with Matrigel (BD Biosciences, San Diego, CA). Then, 600 µL of medium containing 20% FBS was added to the lower chamber. After incubation for 72 h, the cells attached onto the upper side of the transwell were mechanically removed with a cotton stick. Next, the cells on the bottom surface of the membrane were fixed with 4% paraformaldehyde for 10 min and then stained with 0.4% crystal violet solution for 10 min [37]. Images of the migrated and invaded cells were captured with a Nikon Digital Sight DS-L1 camera.

### Detection of ROS levels

Intracellular ROS levels were measured using the ROS Assay Kit (Beyotime, Shanghai, China). Cells were incubated with 10 µM DCFH-DA for 1 h and then treated with the indicated concentrations of H_2_O_2_. The DCF fluorescence intensities were then monitored by flow cytometry [38].

### Kyn level assessment

Kyn levels in the supernatant and cell lysates were measured by ELISA (#ab287800, Abcam) according to the supplier’s instructions [39].

### Cell treatment conditions

NUGC-4 and MKN-45 cells were exposed to the following conditions: control, transfected with empty lentivirus for 48 h; control+kyn, transfected with GPx2 empty lentivirus for 48 h, followed by treatment with 150 µM kyn for 24 h; shGPx2, transfected with GPx2-specific shRNA lentivirus for 48 h; shGPx2+kyn transfected with GPx2-specific shRNA lentivirus for 48 h, followed by treatment with 150 µM kyn for 24 h; H_2_O_2_, transfected with the empty/GPx2-specific shRNA lentivirus for 48 h or treated with H_2_O_2_ (0.01, 0.05, 0.1, 0.5 and 1.0 mM) for 30 min; and NAC, transfected with empty/GPx2-specific shRNA lentivirus or treated with NAC (20 mM) for 1 h.

### Western blotting analysis

Cells were lysed with 1X sodium dodecyl sulfate lysis buffer. Total protein was quantified, separated by SDS‒PAGE, and transferred onto PVDF membranes (Millipore, MA, USA). The target proteins were probed with antibodies against GPx2 (#ab140130, Abcam), KYNU (#11796-1-AP, Proteintech), AhR (#67785-1-Ig, Proteintech), E-cadherin (#3195 S, Cell Signaling Technology), N-cadherin (#13116 S, Cell Signaling Technology), Vimentin (#5741 S, Cell Signaling Technology) and GAPDH (#60004-1-Ig, ProteinTech). Anti-mouse IgG (926-6807, Invitrogen) and anti-rabbit IgG (926-68070, Invitrogen) were used as secondary antibodies. Finally, the protein bands were visualized with enhanced chemiluminescence (ECL; Fdbio Science, Hangzhou, China). Intensity was measured by ImageJ software.

### Subcutaneous xenograft model

To establish the GC xenograft tumor model, 1 × 10^7^ NUGC-4 GC cells in 100 µL of PBS mixed with 100 µL of Matrigel (BD Biosciences) were injected subcutaneously into the right flanks of four-week-old male nude mice. We randomly divided the mice into the control, shGPx2#1 and shGPx2#2 groups (*n* = 5/each group). Mice were monitored for body weight and tumor size (length × width^2^ × 0.5) once a week according to the animal protocol. After 4 weeks, the nude mice were sacrificed, and the tissues were collected for detection [40]. The number of macroscopic nodules was then recorded. Blinding was maintained during the experiments. The protocol was approved by the Committee on the Ethics of Animal Experiments of Zhejiang Chinese Medical University.

### Peritoneal metastasis models

A total of 5 × 10^6^ MKN-45 cells suspended in 200 µL of saline were intraperitoneally injected into five- to six-week-old male nude mice. We randomly and blindly divided the mice into the control, shGPx2#1 and shGPx2#2 groups (*n* = 6/each group). The body weight, living status, and tumor size of the nude mice were recorded. Mice were sacrificed 22 days after GC cell injection, and a small shallow section was cut to expose the abdominal cavity [41]. The number of macroscopic nodules was then recorded. Blinding was maintained during the experiments. The protocol was approved by the Committee on the Ethics of Animal Experiments of Zhejiang Chinese Medical University.

### In vivo luminescence imaging

Mice were anesthetized, and luminescence was measured 5 min after IP injection of D-luciferin sodium salt (150 mg/kg) by using the in vivo imaging system (IVIS) Lumina LT (Caliper Life Sciences, USA). Luciferase activity, which represents the volume of peritoneal metastasis, was measured using the IVIS. Living Image Ver. 4.3 (Caliper Life Sciences, USA) software was used to access the images and acquire the data.

### Statistical analysis

All statistical analyses were performed using GraphPad Prism software version 8.0. Parametric tests (Student’s t-test or one-way ANOVA) or nonparametric tests were used, depending on the type of data distribution and homogeneity of variance. Survival curves were generated using the Kaplan‒Meier method. Count data are presented as the rate or composition ratio using the chi-square test. Data are presented as the mean ± SEM, and *P* < 0.05 was considered to indicate statistical significance.

## Supplementary information

SUPPLEMENTAL MATERIAL

## Data Availability

All data used in the current study are available from the corresponding author on reasonable request.
